# Supplementary material for: Investigating the Effectiveness of Very Low-Calorie Diets and Low-Fat Vegan Diets on Weight and Glycemic Markers in Type 2 Diabetes Mellitus: A Systematic Review and Meta-Analysis
Source: Nutrients. 2022 Nov 17;14(22):4870. doi: 10.3390/nu14224870 (PMC9695880; doi:10.3390/nu14224870)
Supplement: Supplementary file 1 [file nutrients-14-04870-s001.zip › Supplemental Figure S1.pdf]

Supplemental Figure S1

	Vegan			Control				Std. Mean Difference
**Study or Subgroup**	**Mean**	**SD**	**Total**	**Mean**	**SD**	**Total**	**Weight**	**IV, Random, 95% CI**
Lee 2016	23.5	3.4	46	23	2.4	47	27.6%	0.17 [−0.24, 0.58]
Barnard 2006	31.8	7.5	49	34.3	7.3	50	28.4%	−0.34 [−0.73, 0.06]
Barnard 2009	32.3	8.4	49	34.8	7.78	50	28.4%	−0.31 [−0.70, 0.09]
Barnard 2018	32.6	5.67	19	31.5	5.5	21	15.6%	0.19 [−0.43, 0.82]
**Total (95% CI)**			**163**			**168**	**100.0%**	**−0.11 [−0.39, 0.18]**

Heterogeneity: $Tau^2 = 0.03$; $Chi^2 = 4.89$, df = 3 (P = 0.18); $I^2 = 39\%$
Test for overall effect: Z = 0.74 (P = 0.46)

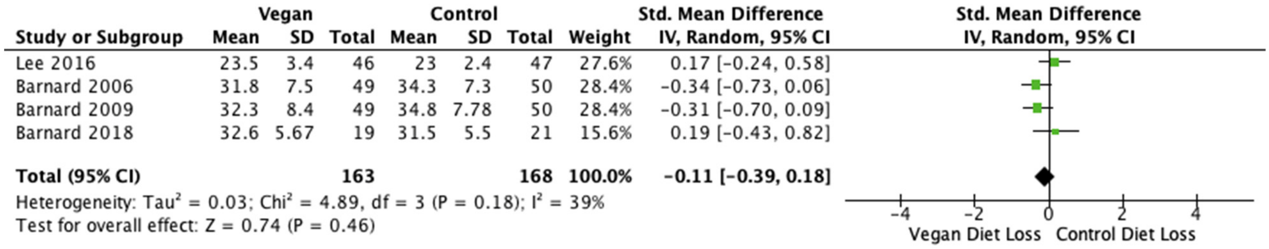

Figure S1. Forest plot comparing the reduction in BMI after consumption of low-fat vegan and control diets.
