# Supplementary material for: Investigating the Effectiveness of Very Low-Calorie Diets and Low-Fat Vegan Diets on Weight and Glycemic Markers in Type 2 Diabetes Mellitus: A Systematic Review and Meta-Analysis
Source: Nutrients. 2022 Nov 17;14(22):4870. doi: 10.3390/nu14224870 (PMC9695880; doi:10.3390/nu14224870)
Supplement: Supplementary file 1 [file nutrients-14-04870-s001.zip › Supplemental Figure S2.pdf]

Supplemental Figure S2

Study or Subgroup	Vegan Mean	Vegan SD	Vegan Total	Control Mean	Control SD	Control Total	Weight	Std. Mean Difference IV, Random, 95% CI
Barnard 2006	1.87	0.63	49	1.5	1.29	50	24.2%	0.36 [−0.04, 0.76]
Barnard 2009	1.29	0.77	49	1.7	2.33	50	24.3%	−0.23 [−0.63, 0.16]
Barnard 2018	1.99	0.94	19	1.02	0.4	21	17.8%	1.34 [0.65, 2.03]
Lee 2016	1.62	1.04	46	1.45	0.65	47	24.0%	0.19 [−0.21, 0.60]
Nicholson 1999	1.87	0.63	7	1.85	1.14	4	9.7%	0.02 [−1.21, 1.25]
**Total (95% CI)**			**170**			**172**	**100.0%**	**0.32 [−0.15, 0.78]**

Heterogeneity: $Tau^2 = 0.19$; $Chi^2 = 15.68$, df = 4 (P = 0.003); $I^2 = 74\%$

Test for overall effect: Z = 1.34 (P = 0.18)

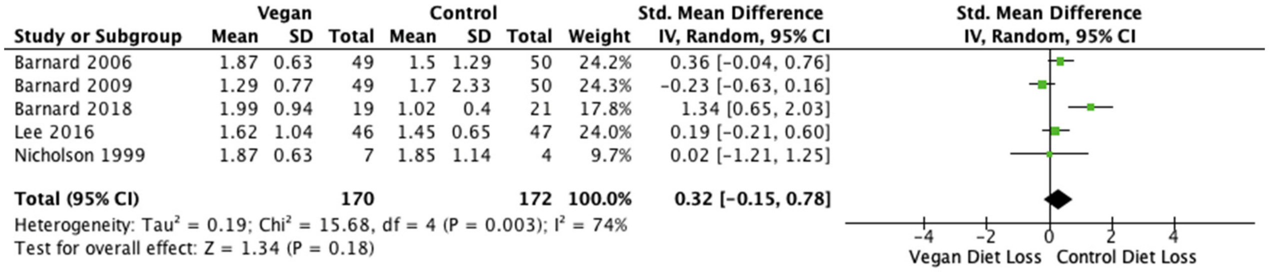

Figure S2. Forest plot comparing the reduction in triglyceride levels after consumption of low-fat vegan and control diets.
