# Supplementary material for: Investigating the Effectiveness of Very Low-Calorie Diets and Low-Fat Vegan Diets on Weight and Glycemic Markers in Type 2 Diabetes Mellitus: A Systematic Review and Meta-Analysis
Source: Nutrients. 2022 Nov 17;14(22):4870. doi: 10.3390/nu14224870 (PMC9695880; doi:10.3390/nu14224870)
Supplement: Supplementary file 1 [file nutrients-14-04870-s001.zip › Supplemental Figure S3.pdf]

Supplemental Figure S3

Study or Subgroup	Vegan Mean	Vegan SD	Vegan Total	Control Mean	Control SD	Control Total	Weight	Std. Mean Difference IV, Random, 95% CI
Barnard 2006	105.5	18.1	49	109.5	14.7	50	34.0%	−0.24 [−0.64, 0.15]
Barnard 2009	106.6	19.6	49	110.5	2.1	50	33.9%	−0.28 [−0.68, 0.12]
Lee 2016	81.9	7.5	46	81.5	7.9	47	32.1%	0.05 [−0.36, 0.46]
**Total (95% CI)**			**144**			**147**	**100.0%**	**−0.16 [−0.39, 0.07]**

Heterogeneity: $Tau^2 = 0.00$; $Chi^2 = 1.55$, df = 2 (P = 0.46); $I^2 = 0\%$
Test for overall effect: Z = 1.36 (P = 0.17)

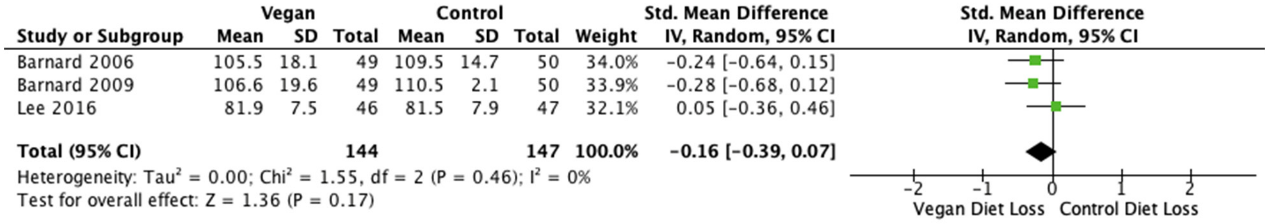

Figure S3. Forest plot comparing the reduction in waist circumference after consumption of low-fat vegan and control diets.
