# Supplementary material for: Investigating the Effectiveness of Very Low-Calorie Diets and Low-Fat Vegan Diets on Weight and Glycemic Markers in Type 2 Diabetes Mellitus: A Systematic Review and Meta-Analysis
Source: Nutrients. 2022 Nov 17;14(22):4870. doi: 10.3390/nu14224870 (PMC9695880; doi:10.3390/nu14224870)
Supplement: Supplementary file 1 [file nutrients-14-04870-s001.zip › Supplemental Figure S4.pdf]

Supplemental Figure S4

	Vegan			Control				Std. Mean Difference
Study or Subgroup	Mean	SD	Total	Mean	SD	Total	Weight	IV, Random, 95% CI
Barnard 2006	4.12	0.83	49	4.52	0.94	50	35.0%	−0.45 [−0.85, −0.05]
Barnard 2009	4.28	0.84	49	4.76	0.99	50	34.9%	−0.52 [−0.92, −0.12]
Barnard 2018	3.79	0.75	19	3.54	0.74	21	22.1%	0.33 [−0.30, 0.95]
Nicholson 1999	4.63	1.32	7	4.93	0.46	4	7.9%	−0.25 [−1.48, 0.99]
**Total (95% CI)**			**124**			**125**	**100.0%**	**−0.28 [−0.66, 0.09]**

Heterogeneity: $Tau^2 = 0.06$; $Chi^2 = 5.46$, df = 3 (P = 0.14); $I^2 = 45\%$
Test for overall effect: Z = 1.49 (P = 0.14)

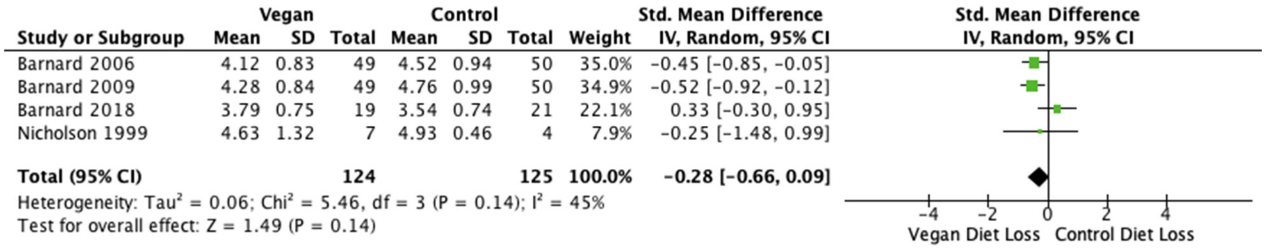

Figure S4. Forest plot comparing the reduction in total cholesterol after consumption of low-fat vegan and control diets.
