# Supplementary material for: Investigating the Effectiveness of Very Low-Calorie Diets and Low-Fat Vegan Diets on Weight and Glycemic Markers in Type 2 Diabetes Mellitus: A Systematic Review and Meta-Analysis
Source: Nutrients. 2022 Nov 17;14(22):4870. doi: 10.3390/nu14224870 (PMC9695880; doi:10.3390/nu14224870)
Supplement: Supplementary file 1 [file nutrients-14-04870-s001.zip › Supplemental Figure S5.pdf]

Supplemental Figure S5

	Vegan			Control				Std. Mean Difference
**Study or Subgroup**	**Mean**	**SD**	**Total**	**Mean**	**SD**	**Total**	**Weight**	**IV, Random, 95% CI**
Barnard 2006	1.22	0.44	49	1.21	0.31	50	29.1%	0.03 [−0.37, 0.42]
Barnard 2009	1.33	0.49	49	1.26	0.35	50	29.0%	0.16 [−0.23, 0.56]
Barnard 2018	1.32	0.45	19	1.46	0.4	21	11.6%	−0.32 [−0.95, 0.30]
Lee 2016	1.35	0.39	46	1.34	0.34	47	27.4%	0.03 [−0.38, 0.43]
Nicholson 1999	0.95	0.28	7	1.1	0.17	4	2.8%	−0.55 [−1.81, 0.71]
**Total (95% CI)**			**170**			**172**	**100.0%**	**0.01 [−0.20, 0.22]**

Heterogeneity: $Tau^2 = 0.00$; $Chi^2 = 2.45$, df = 4 (P = 0.65); $I^2 = 0\%$
Test for overall effect: Z = 0.09 (P = 0.93)

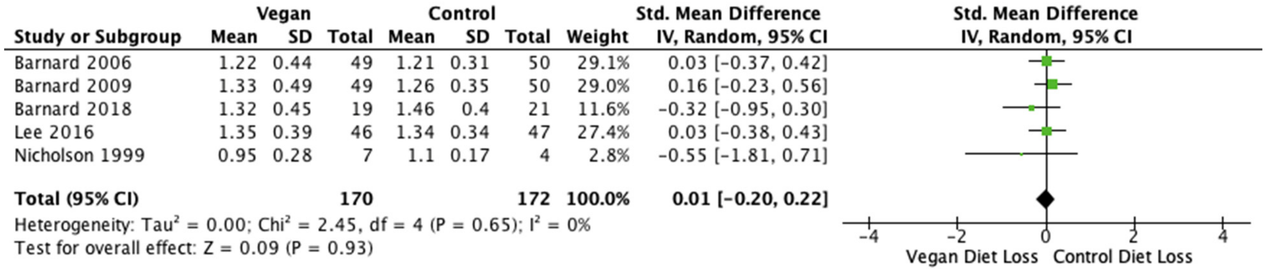

Figure S5. Forest plot showing mean HDL cholesterol after consumption of low-fat vegan and control diets.
