# Supplementary material for: Investigating the Effectiveness of Very Low-Calorie Diets and Low-Fat Vegan Diets on Weight and Glycemic Markers in Type 2 Diabetes Mellitus: A Systematic Review and Meta-Analysis
Source: Nutrients. 2022 Nov 17;14(22):4870. doi: 10.3390/nu14224870 (PMC9695880; doi:10.3390/nu14224870)
Supplement: Supplementary file 1 [file nutrients-14-04870-s001.zip › Supplemental Figure S6.pdf]

Supplemental Figure S6

Study or Subgroup	VLCD Mean	VLCD SD	VLCD Total	Non-Dietary Therapies Mean	Non-Dietary Therapies SD	Non-Dietary Therapies Total	Weight	Std. Mean Difference IV, Random, 95% CI
Taheri 2020	1.71	1.03	70	1.58	0.86	77	37.8%	0.14 [−0.19, 0.46]
Williams 1998	1.31	0.875	31	1.89	1.01	16	32.4%	−0.62 [−1.24, −0.00]
Wing 1991	1.61	0.65	17	1.02	0.32	16	29.8%	1.11 [0.37, 1.85]
**Total (95% CI)**			**118**			**109**	**100.0%**	**0.18 [−0.61, 0.97]**

Heterogeneity: $Tau^2 = 0.40$; $Chi^2 = 12.42$, df = 2 (P = 0.002); $I^2 = 84\%$
Test for overall effect: Z = 0.45 (P = 0.65)

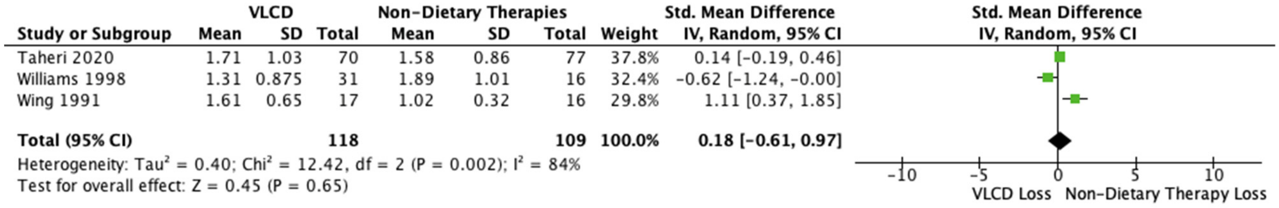

Figure S6. Forest plot comparing the reduction in triglycerides after VLCD diets and non-dietary therapies.
