# Supplementary material for: Investigating the Effectiveness of Very Low-Calorie Diets and Low-Fat Vegan Diets on Weight and Glycemic Markers in Type 2 Diabetes Mellitus: A Systematic Review and Meta-Analysis
Source: Nutrients. 2022 Nov 17;14(22):4870. doi: 10.3390/nu14224870 (PMC9695880; doi:10.3390/nu14224870)
Supplement: Supplementary file 1 [file nutrients-14-04870-s001.zip › Supplemental Figure S7.pdf]

Supplemental Figure S7

	VLCD			Non-Dietary Therapies				Std. Mean Difference
Study or Subgroup	Mean	SD	Total	Mean	SD	Total	Weight	IV, Random, 95% CI
Taheri 2020	5.15	1.05	70	4.22	0.94	77	38.1%	0.93 [0.59, 1.27]
Williams 1998	5.125	1.195	31	5.21	1.06	16	31.9%	-0.07 [-0.68, 0.53]
Wing 1991	4.63	0.86	17	4.5	1.17	16	29.9%	0.12 [-0.56, 0.81]
**Total (95% CI)**			**118**			**109**	**100.0%**	**0.37 [-0.33, 1.07]**

Heterogeneity: $Tau^2 = 0.30$; $Chi^2 = 10.25$, df = 2 (P = 0.006); $I^2 = 80\%$
Test for overall effect: Z = 1.04 (P = 0.30)

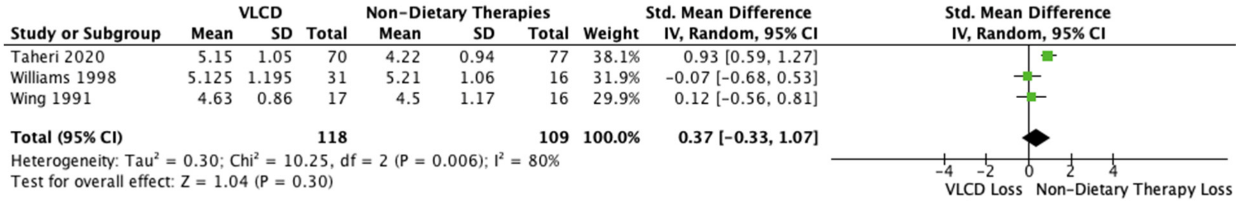

Figure S7. Forest plot comparing the reduction in total cholesterol after VLCD diets and non-dietary therapies.
