# Supplementary material for: Investigating the Effectiveness of Very Low-Calorie Diets and Low-Fat Vegan Diets on Weight and Glycemic Markers in Type 2 Diabetes Mellitus: A Systematic Review and Meta-Analysis
Source: Nutrients. 2022 Nov 17;14(22):4870. doi: 10.3390/nu14224870 (PMC9695880; doi:10.3390/nu14224870)
Supplement: Supplementary file 1 [file nutrients-14-04870-s001.zip › Supplemental Figure S8.pdf]

Supplemental Figure S8

Study or Subgroup	VLCD Mean	VLCD SD	VLCD Total	Non-Dietary Therapies Mean	Non-Dietary Therapies SD	Non-Dietary Therapies Total	Weight	Std. Mean Difference IV, Random, 95% CI
Wing 1991	104	88	17	120	62	16	43.7%	−0.20 [−0.89, 0.48]
Williams 1998	103.5	43	31	106	55	16	56.3%	−0.05 [−0.66, 0.55]
**Total (95% CI)**			**48**			**32**	**100.0%**	**−0.12 [−0.57, 0.33]**

Heterogeneity: $Tau^2 = 0.00$; $Chi^2 = 0.11$, df = 1 (P = 0.74); $I^2 = 0\%$
Test for overall effect: Z = 0.51 (P = 0.61)

Std. Mean Difference
IV, Random, 95% CI

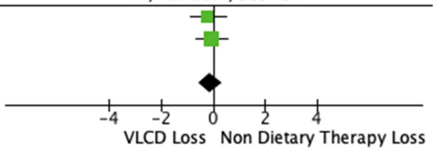

Figure S8. Forest plot showing mean fasting insulin levels after VLCD and non-dietary therapies.
