# Supplementary material for: Investigating the Effectiveness of Very Low-Calorie Diets and Low-Fat Vegan Diets on Weight and Glycemic Markers in Type 2 Diabetes Mellitus: A Systematic Review and Meta-Analysis
Source: Nutrients. 2022 Nov 17;14(22):4870. doi: 10.3390/nu14224870 (PMC9695880; doi:10.3390/nu14224870)
Supplement: Supplementary file 1 [file nutrients-14-04870-s001.zip › Supplemental Figure S9.pdf]

Supplemental Figure S9

	VLCD			Non-Dietary Therapies				Std. Mean Difference	Std. Mean Difference
Study or Subgroup	Mean	SD	Total	Mean	SD	Total	Weight	IV, Random, 95% CI	IV, Random, 95% CI
Taheri 2020	1.15	0.27	70	1	0.23	77	38.5%	0.60 [0.27, 0.93]	
Williams 1998	1.105	0.225	31	1.05	0.3	16	32.0%	0.21 [−0.39, 0.82]	
Wing 1991	1.07	0.27	17	1.26	0.3	16	29.5%	−0.65 [−1.35, 0.05]	
**Total (95% CI)**			**118**			**109**	**100.0%**	**0.11 [−0.59, 0.80]**	

Heterogeneity: $Tau^2 = 0.30$; $Chi^2 = 10.12$, df = 2 (P = 0.006); $I^2 = 80\%$
Test for overall effect: Z = 0.30 (P = 0.76)

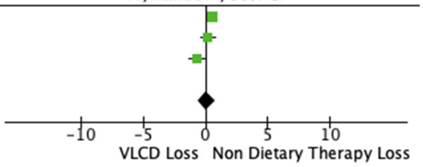

Figure S9. Forest plot showing mean HDL cholesterol VLCD and non-dietary therapies.
