# Supplementary material for: Investigating the Effectiveness of Very Low-Calorie Diets and Low-Fat Vegan Diets on Weight and Glycemic Markers in Type 2 Diabetes Mellitus: A Systematic Review and Meta-Analysis
Source: Nutrients. 2022 Nov 17;14(22):4870. doi: 10.3390/nu14224870 (PMC9695880; doi:10.3390/nu14224870)
Supplement: Supplementary file 1 [file nutrients-14-04870-s001.zip › Table S1 updated.pdf]

Table S1. Study Characteristics of Included Studies.

Study	Country	Study Design	Study Duration		Intervention diet (I)	Control diet (C) or not under review	Number of participants All (I / C)	M/F	Mean Age, years (I / C)
			Intervention	Follow-up					
Barnard et al. (2006)	USA	RCT	22 weeks	22 weeks	LFVD	ADA Diet	99 (49 / 50)	M/F	55.65 (56.7 / 54.6)
Barnard et al. (2009)	USA	RCT	74 weeks	74 weeks	LFVD	ADA Diet	99 (49 / 50)	M/F	55.65 (56.7 / 54.6)
Barnard et al. (2018)	USA	RCT	20 weeks	20 weeks	LFVD	Portion Controlled Diet	40 (19 / 21)	M/F	61 (61 / 61)
Jackness et al. (2013)	USA	NRCT	21 days	21 days	VLCD (500kcal/day)	Roux-En-Y Gastric Bypass (RYGB)	25 (14 / 11)	NI	48.25 (51.9 / 44.6)
Jazet et al. (2007)	The Netherlands	BAS	17 weeks	17 weeks	VLCD (450 kcal/day)	No Dietary Intervention	10 (10[1])	M/F	55
Lee et al. (2016)	Korea	RCT	12 weeks	12 weeks	LFVD	KDA Diet	93 (47 / 46)	M/F	57.9 (57.5 / 58.3)
Lim et al. (2011)	UK	SAIT	8 weeks	12 weeks	VLCD (600 kcal/day)	No Dietary Intervention	20 (11 / 9)	M/F	49.6 (49.5 / 49.7)
Nicholson et al. (1999)	USA	RCT	12 weeks	12 weeks	LFVD	Conventional LF Diet	11 (7 / 4)	M/F	55.75 (51 / 60)
Skrha et al. (2005)	Czech Republic	NRPS	8 days	8 days	VLCD (600 kcal/day)	VLCD (600 kcal/day)	18 (9 / 9)	M/F	53.5 (55 / 52)
Snel et al. (2011)	Belgium	RCT	4 months	18 months	VLCD (450 kcal/day)	VLCD (450 kcal/day) + Exercise program	139 (27 / 112)	M/F	57.5 (VLCD = 59, VLCD + Exercise = 56 / Lean controls = 59, Obese controls = 56)
Snel et al. (2012)	Belgium	BAS	18 months	18 months	VLCD	N/A	14 (14[1])	M/F	53
Steven + Taylor (2015)	UK	SAIT	8 weeks	8 weeks	VLCD (624 kcal/day)	N/A	29 (29[1])	M/F	56.85 (52.1 / 61.6)
Taheri et al. (2020)	Qatar	RCT	12 months	12 months	VLCD (800 kcal/day)	Standard medical care	147 (70 / 77)	M/F	42.1 (41.9 / 42.3)
Teeuwisse et al. (2012)	The Netherlands	BAS	4 days	4 days	VLCD (450 kcal/day)	N/A	10 (10[1])	M	56.8
Williams et al. (1998)	USA	RCT	20 weeks	20 weeks	VLCD (400-600 kcal/day)	SBTP (1500-1800kcal/day)	47 (31 / 16)	M/F	51.9 (Control =51.4, 5 Day Intervention= 50.3, 1 Day Intervention =54.1
Wing et al. (1991)	USA	RCT	20 weeks	72 weeks	VLCD (401 kcal/day)	SBTP (1003-1505kcal/day)	33 (17 / 16)	M/F	51.25 (50.6 / 51.9)

[1] Intervention diet only

ADA, American diabetes association; BAS, Before-after study; C, Control; I, Intervention; KDA, Korean diabetes association; LF, Low-fat; LFVD, Low-fat vegan diet; NI, No Information; NRCT, Non-randomized control trial; NRPS, Non-Randomized Pilot Study; RCT, Randomized control trial; SAIT, Single arm intervention trial; SBTP, Standard behavior therapy program; VLCD, Very low-calorie diet
